# Resveratrol Inhibits Sodium/Iodide Symporter Gene Expression and Function in Rat Thyroid Cells

**DOI:** 10.1371/journal.pone.0107936

**Published:** 2014-09-24

**Authors:** Cesidio Giuliani, Ines Bucci, Serena Di Santo, Cosmo Rossi, Antonino Grassadonia, Marianna Mariotti, Mauro Piantelli, Fabrizio Monaco, Giorgio Napolitano

**Affiliations:** 1 Unit of Endocrinology, Department of Medicine and Sciences of Aging, “G. D'Annunzio” University of Chieti–Pescara, Chieti, Italy; 2 Aging Research Centre (Ce.S.I.), “G. D'Annunzio” University Foundation, Chieti, Italy; 3 Department of Oncology and Neurosciences, “G. D'Annunzio” University of Chieti–Pescara, Chieti, Italy; Institute of Experimental Endocrinology and Oncology 'G. Salvatore' (IEOS), Italy

## Abstract

Resveratrol is a polyphenol found in grapes and berries that has antioxidant, antiproliferative and anti-inflammatory properties. For these reasons, it is available as a dietary supplement, and it is under investigation in several clinical trials. Few data are available regarding the effects of resveratrol on thyroid function. A previous study showed that resveratrol transiently increases iodide influx in FRTL-5 rat thyroid cells. Indeed, this increase arises after short treatment times (6–12 h), and no further effects are seen after 24 h. The aim of the present study was to investigate the effects of resveratrol on iodide uptake and sodium/iodide symporter expression in thyroid cells after longer times of treatment. For this purpose, the effects of resveratrol were evaluated both *in vitro* and *in vivo* using the rat thyroid FRTL-5 cell line and Sprague-Dawley rats, respectively. In FRTL-5 cells, resveratrol decreased the sodium/iodide symporter RNA and protein expression as a function of time. Furthermore, resveratrol decreased cellular iodide uptake after 48 h of treatment. The inhibitory effect of resveratrol on iodide uptake was confirmed *in vivo* in Sprague-Dawley rats. This study demonstrates that with longer-term treatment, resveratrol is an inhibitor of sodium/iodide symporter gene expression and function in the thyroid. These data suggest that resveratrol can act as a thyroid disruptor, which indicates the need for caution as a supplement and in therapeutic use.

## Introduction

Resveratrol (3,4′,5-trihydroxystilbene) is a natural polyphenol that is present in grapes, berries, peanuts and other plants, where it acts as a phytoalexin, to protect the plant from pathogenic attack [1]. Several studies have shown that resveratrol has many therapeutically relevant properties, such as antioxidant, anti-inflammatory and antiproliferative activities [1]–[4]. For these reasons, there is great interest for the use of resveratrol in several chronic human diseases, such as cancer and diabetes, and in neurodegenerative and cardiovascular disorders. Indeed, resveratrol is available as a dietary supplement, and its use is being investigated for various disorders in several clinical trials, both completed and ongoing [5]–[7].

With respect to the thyroid, few data are available on the effects of resveratrol on thyroid function [8]. Some reports have shown an antiproliferative effect of resveratrol in thyroid cancer cell lines [9]–[11]. A recent study showed that can resveratrol also increase the expression of some thyroid-specific genes, including the sodium/iodide symporter (NIS) gene, in human thyroid anaplastic carcinoma cell lines [11].

To the best of our knowledge, there is only one report that used a nontransformed thyroid cell line to evaluate the effects of resveratrol on thyroid cells [12]. This report showed that resveratrol increases NIS protein expression and iodide uptake after 6 h to 12 h of treatment, and that this increase was transient, as it was no longer detectable after 24 h. However, they provided no data on the effects of resveratrol on NIS RNA [12].

Given these data, we studied on the effects of resveratrol on normal thyroid cells as a function of time, and we show that after 48 h of treatment, resveratrol down-regulates the expression of the NIS gene and inhibits iodide uptake. Furthermore, we investigated the *in-vivo* effects of resveratrol on thyroid radioiodine uptake in Sprague-Dawley rats. These treatments with resveratrol significantly decreased the thyroid radioiodine uptake, in comparison with the control vehicle, thus confirming the *in-vitro* data.

These data indicate that resveratrol is an inhibitor of NIS expression and function in normal thyroid cells. Furthermore, resveratrol appears to have a role as a thyroid disruptor, and thus we suggest caution with ingestion of large amounts of resveratrol. Further studies are required to confirm these data in human.

## Materials and Methods

### Materials

Resveratrol was from Sigma-Aldrich Co (St. Louis, MO, USA). Heat-treated, mycoplasma-free calf serum was from Life Technologies Europe (Monza, Italy). [α-^32^P]-dCTP and [^125^I]-NaI were from Perkin Elmer Italia (Monza, Italy). The source of all of the other materials was Sigma-Aldrich, unless otherwise specified.

### Cell culture

The F1 subclone of FRTL-5 rat thyroid cells (American Type Culture Collection, CRL-8305) was a gift from the Interthyr Research Foundation (Woodinville, WA, USA). These FRTL-5 cells were grown in six-hormone (6H) medium of Coon's modified Ham's F-12 supplemented with 5% calf serum, 2 mM glutamine, 1 mM nonessential amino acids, and the 6H mixture (6H5% medium): bovine thyroid-stimulating hormone (TSH; 1 mU/ml), insulin (10 µg/ml), cortisol (0.4 ng/ml), transferrin (5 µg/ml), glycyl-L-histidyl-L-lysine acetate (10 ng/ml), and somatostatin (10 ng/ml). The cells were diploid, between the 5^th^ and 25^th^ passage, and had all of the functional properties described previously [13]–[20]. Fresh 6H5% medium was added every 2 to 3 days, and the cells were passaged every 7 days. In individual experiments, the cells were transferred to a five-hormone (5H) medium (i.e., without TSH), again with 5% calf serum (5H5% medium), as described below.

In all of the experiments with resveratrol, the medium was changed every 24 h, with the addition of fresh medium with resveratrol. Resveratrol was taken from an absolute ethanol stock solution, with control cells treated with the same amount of vehicle. The final ethanol concentration was thus identical in the control and treated samples, and did not exceed 0.5% (v/v).

### RNA isolation and Northern analysis

The FRTL-5 cells were grown to 60% confluency in 6H5% medium, and then maintained in 5H5% medium for 6 days, as indicated above. In individual experiments, the cells kept in 5H5% medium were shifted to 6H5% medium for 24 h before treatment with control vehicle or resveratrol for the indicated times. RNA was prepared using RNeasy Mini kits (Qiagen Inc., Valencia, CA, USA). Twenty µg of the different RNA samples were run on denaturing agarose gels, capillary blotted on Nytran membranes (Schleicher & Schuell-Whatman, Florrham Park, NJ, USA), UV cross-linked, and hybridized using QuickHyb Hybridization Solution (Stratagene, La Jolla, CA, USA), following the manufacturer protocol. The probes were labeled with [α-^32^P]-dCTP using Ladderman Labeling kits (Takara Mirus Bio, Madison, WI, USA). The NIS and β-actin probes were as described previously [17], [19]. Quantification was performed using a STORM 860 Imager (Molecular Dynamics, GE Healthcare Italy, Milan, Italy).

### Whole-cell extracts and Western blotting

To prepare whole-cell lysates, the FRTL-5 cells were collected, washed with ice-cold phosphate-buffered saline, and resuspended in ice-cold RIPA lysis buffer (Sigma-Aldrich). The cells were incubated on ice for 15 min before being vortexed. After centrifugation to remove the cell debris, the cell lysates were subjected to 10% SDS-PAGE, and the proteins separated were transferred to nitrocellulose membranes by electrophoretic blotting. After this transfer, the membranes were incubated according to the manufacturer instructions, using a mouse monoclonal anti-NIS antibody (NBP1-70342, Novus Biologicals Europe, Cambridge, UK). The membranes were subsequently washed and incubated with a horseradish peroxidase-conjugated anti-mouse secondary antibody (ab6789, Abcam, Cambridge, UK), following the manufacturer instructions. The proteins were detected with ECL plus (GE Healthcare). Quantitation was performed using the STORM 860 Imager (Molecular Dynamics, GE Healthcare).

### Measurement of iodide uptake

Uptake of [^125^I^-^] by the FRTL-5 cells was measured as previously described [20]. Briefly, the cells were seeded in 12-well plates, grown to 60% confluency in 6H5% medium, and shifted to 5H5% medium (i.e., without TSH) for 6 days. Then TSH (1 mU/ml) was added with the control vehicle or with resveratrol, for the indicated times. After the treatments, the cells were washed twice with 1 ml HEPES-buffered modified Hank's balanced salt solution (HBSS) (137 mM NaCl, 5.4 mM KCl, 1.3 mM CaCl_2_, 0.4 mM MgSO_4_, 0.5 mM Na_2_HPO_4_, 0.44 mM KH_2_PO_4_, 5.55 mM glucose, 10 mM HEPES, pH 7.3) and incubated for 40 min at 37°C in 250 µl modified HBSS containing 0.5 µCi carrier-free [^125^I]-NaI and 30 µM NaI. The cells were then washed twice with ice-cold modified HBSS. Absolute ethanol (500 µl) was added to each well for 30 min, and 50 µl of the well contents were transferred into scintillation vials and counted in a gamma-counter. Iodide uptake was evaluated as picomoles per µg DNA.

### Animals and radioiodine uptake

All of the experiments performed on animals were carried out in accordance with the European Union Directive 2010/63/EU for animal experiments, and were approved by the Interuniversity Animal Research Ethics Committee of the Chieti–Pescara and Teramo Universities (CEISA, Italy; prot.12/2013/CEISA/COMM/PROG.47). Twelve male adult Sprague-Dawley rats from our breeding colony (age, 8 weeks; weight, 250 g) were housed in a temperature-controlled room with the standard light and dark cycles, with food pellets and water *ad libitum*. The rats were treated daily for 14 days, by intraperitoneal (i.p.) injection of the control vehicle or of resveratrol 50 mg/kg, suspended by sonication in phosphate-buffered saline containing 20% polyethylene glycol 400 and 2% Tween-80. On the last day of treatment, each rat received 185 kBq [^125^I]-NaI diluted in sterile saline, by i.p. injection. After 24 h, the rats were sacrificed using carbon dioxide narcosis, their thyroid glands were removed, and the radioiodine uptake was evaluated using a gamma-counter (Packard Cobra II auto-gamma, Perkin Elmer). The data are evaluated as % [^125^I] uptake/mg thyroid gland.

### Immunofluorescence analysis

Eight male adult Sprague-Dawley rats from our breeding colony (age, 8 weeks; weight, 250 g) were treated with resveratrol 50 mg/kg or the control vehicle for 14 days, as described in the previous section for the radioiodine uptake. After euthanasia, the thyroid glands were removed, mounted in Optimal Cutting Temperature compound (Bio-Optica, Milan, Italy), and frozen immediately in liquid nitrogen. Sections of 6 µm were fixed with cold 100% acetone at 4°C for 10 min, and incubated with a mouse monoclonal anti-NIS antibody (NBP1-70342, Novus Biologicals Europe) at a 1∶50 dilution, overnight at 4°C. After washing, the sections were incubated with an anti-mouse fluorescein-conjugated secondary antibody (Alexa Fluor 488, Life Technologies) at a dilution of 1∶200 for 1 h at room temperature. Po-Pro-3 iodide (Life Technologies) was used at a dilution of 1∶1000 for 1 h at room temperature, to stain nuclei. Sections incubated with mouse IgG from Vector Laboratories (Burlingame, Ca, USA) instead of the primary antibody served as a negative control. The slides were visualized under a Zeiss LSM S10 confocal microscope (Carl Zeiss SpA, Milan, Italy), with a x40 immersion lens.

### Other assays

Protein concentrations were determined using BCA protein assays kits (Pierce Biotechnology Inc, Rockford, IL, USA), with crystalline bovine serum albumin as the standard.

### Statistical analysis

The data are expressed as means ±standard deviation (±SD). The significance between the experimental values was determined by two-way analysis of variance. Differences were considered significant when P<0.05.

## Results

### Resveratrol down-regulates NIS gene expression in FRTL-5 cells

**Figure 1:**
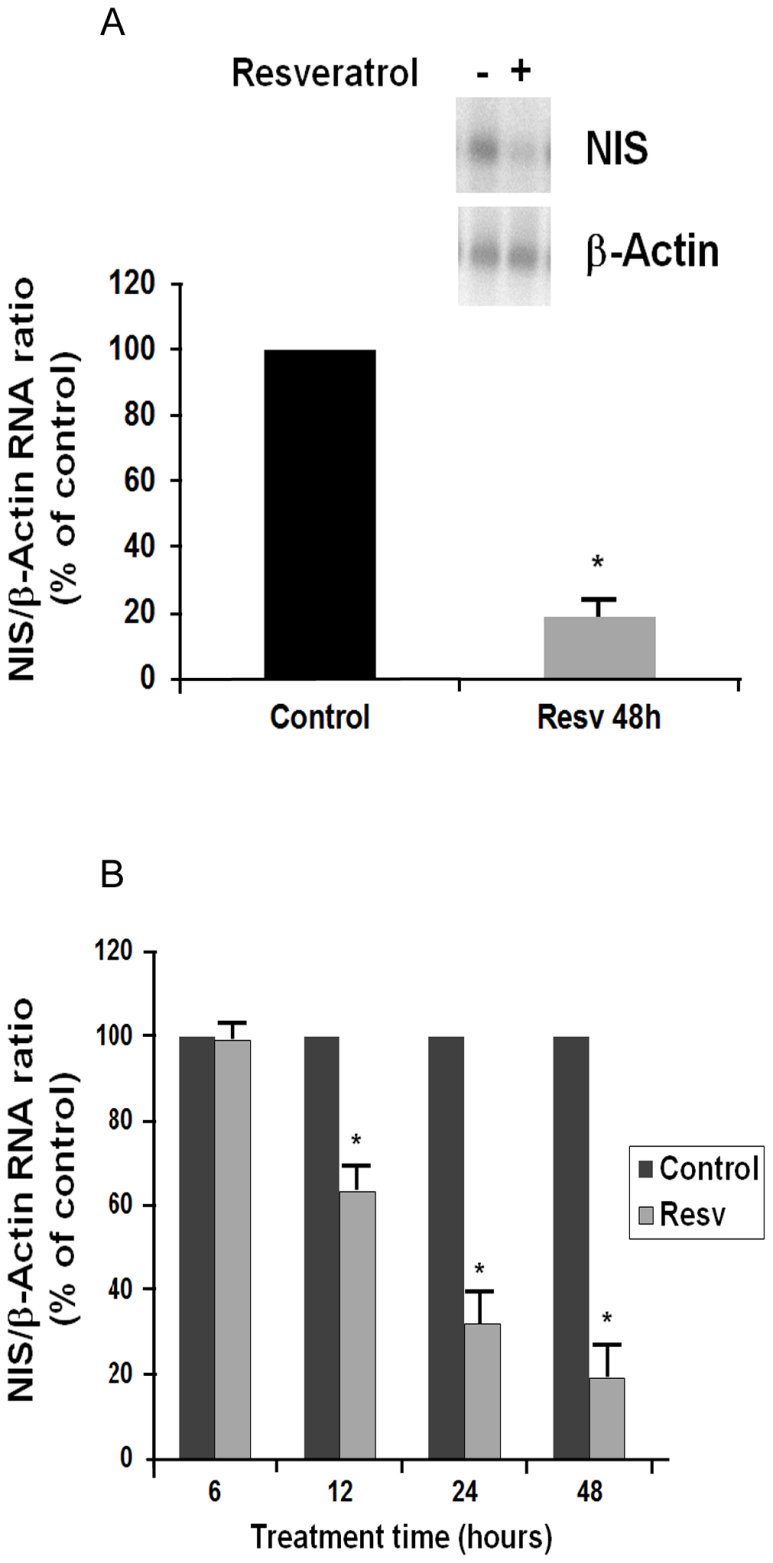
Effect of 10 µM resveratrol on NIS gene and β-actin RNA levels. FRTL-5 cells were grown to 60% confluency in 6H5% medium, and then shifted to 5H5% medium for 6 days, before being cultured again in 6H5% medium for 24 h, and finally treated with resveratrol. A, Effects of 10 µM resveratrol after 48 h of treatment. Insert: Representative Northern blot. B, Effects of resveratrol 10 µM as a function of time. Data are the normalized means ±SD (against β-actin) from three independent experiments (control vehicle: 100%). Control, cells treated with the control vehicle (0.5% ethanol); Resv, cells treated with 10 µM resveratrol. *, p<0.05 *versus* relevant control.

The FRTL-5 cells were grown in 6H5% medium until 60% confluent, and then switched to 5H5% medium (i.e., without TSH) for 6 days, to become quiescent; they were then cultured again in 6H5% medium for 24 h, and then treated with resveratrol at the doses and times indicated. As shown in Figure 1A, treatment with 10 µM resveratrol for 48 h significantly decreased NIS RNA expression, to 19%±3% of the control. This resveratrol effect was concentration and time dependent. After 48 h treatment, there was a significant decrease in NIS RNA using 5 µM resveratrol (65%±4% of the control), with further suppression at 10 µM resveratrol (19%±3% of the control). The time-course of this action showed a significant decrease in NIS RNA after 12 h with 10 µM resveratrol (63%±7% of the control), which increased further to 48 h (Fig. 1B).

**Figure 2:**
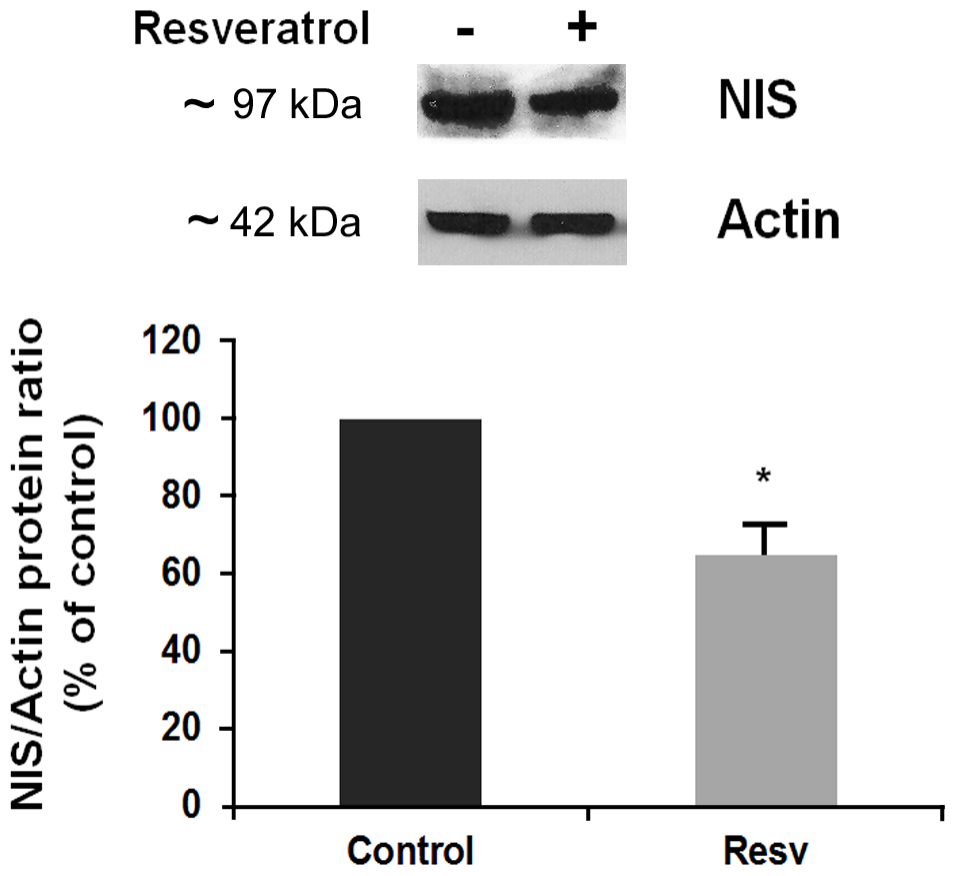
Effects of 10 µM resveratrol on NIS protein expression. FRTL-5 cells were grown to 60% confluency in 6H5% medium, and then shifted to 5H5% medium for 6 days, before being cultured again in 6H5% medium for 24 h, and finally treated with resveratrol for 48 h. A representative Western blot is shown (top), with quantification (bottom). Data are the normalized means ±SD (against actin) from three independent experiments (control vehicle: 100%). Control, cells treated with the control vehicle (0.5% ethanol); Resv, cells treated with 10 µM resveratrol. *, p<0.05 *versus* relevant control.

**Figure 3:**
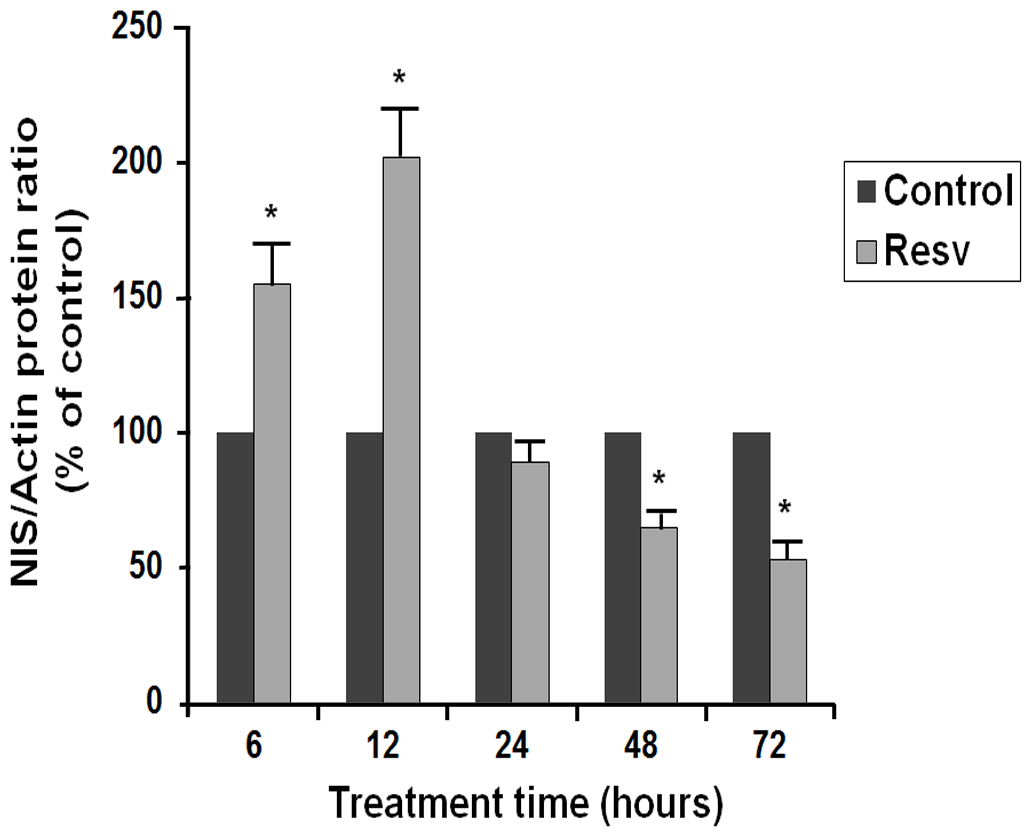
Effects of 10 µM resveratrol on NIS protein expression as a function of time. FRTL-5 cells were grown in 6H5% medium (containing TSH 1 mU/ml) and treated for the indicated time with 10 µM resveratrol. Data for quantification following Western blotting are the normalized means ±SD (against actin) from three independent experiments (control vehicle: 100%). Control, cells treated with the control vehicle (0.5% ethanol); Resv, cells treated with 10 µM resveratrol. * p<0.05 *versus* relevant controls.

These inhibitory effects of resveratrol were confirmed in the evaluation of the NIS protein expression, using Western blotting. The FRTL-5 cells were cultured as described above for the RNA experiments, and treated with 10 µM resveratrol. As shown in Figure 2, this resveratrol treatment significantly decreased the NIS protein expression after 48 h, to 65%±5% of the control. Given the contradictory results observed previously [12], we performed a time-course. FRTL-5 cells maintained in their standard 6H medium containing 1 mU/ml TSH were treated with resveratrol 10 µM at the times indicated. As shown in Figure 3, resveratrol caused an increase in the NIS protein expression after 6 h and 12 h of treatment, confirming the observation previously reported [12]. However, this increase was transient and was followed by a significant reduction in NIS protein expression after 48 h and 72 h of treatment (Fig. 3).

### Down-regulation of the NIS gene expression by resveratrol results in decreased iodide uptake in FRTL-5 cells

**Figure 4:**
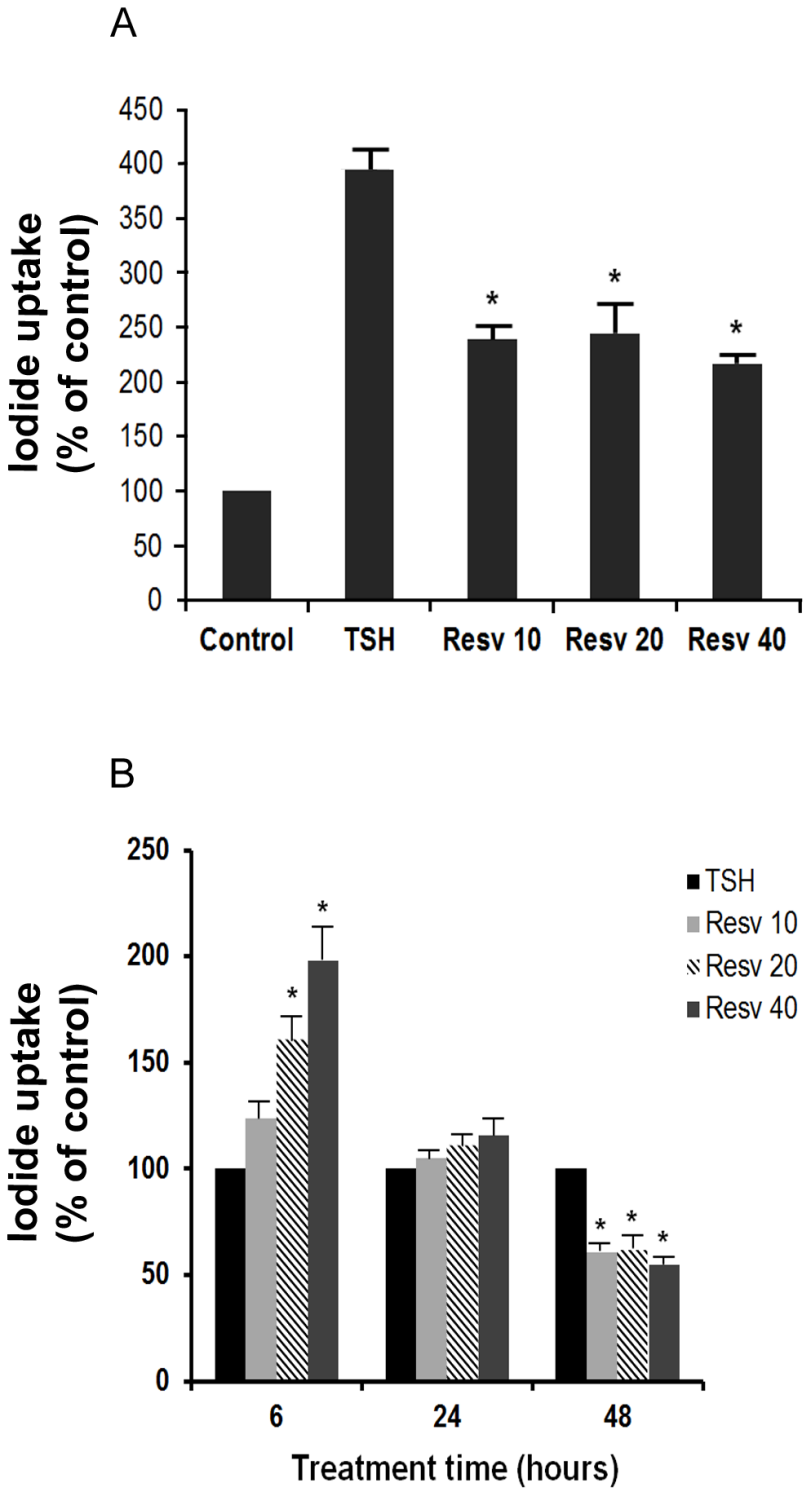
Effects of resveratrol on TSH induction of iodide uptake. FRTL-5 cells were grown in 12-well plates to 60% confluency in 6H5% medium, and then shifted to 5H5% medium for 6 days. TSH (1 mU/ml) was then added without and with resveratrol. A, Effects of resveratrol as a function of concentration after 48 h of treatment. B, Effect of resveratrol as a function of time. Data (from pmole per µg DNA) are normalized means ±SD from three independent experiments (control vehicle, 100%). Control, cells maintained in 5H5% medium (without TSH); TSH, cells with 1 mU/ml TSH + control vehicle (0.5% ethanol); Resv 10, 20, 40, cells with TSH 1 mU/ml + 10, 20, 40 µM resveratrol, respectively. * p<0.05 *versus* relevant control.

We next investigated whether the effects of resveratrol on NIS expression are associated with decreased activity of iodide uptake. The FRTL-5 cells were grown in 6H5% medium until 60% confluent, then switched to 5H5% medium (i.e., without TSH) for 6 days. This resulted in a complete loss of iodide from these cells, and very low levels of NIS RNA expression and NIS protein production [20], [21]. The cells were then treated with 1 mU/ml TSH, and the vehicle control (0.5% ethanol) or 10 µM resveratrol for 48 h, with the iodide uptake measured using 0.5 µCi carrier-free [^125^I]-NaI and 30 µM NaI, as described in the Materials and methods. As shown in Figure 4A, the addition of resveratrol significantly decreased the TSH induction of iodide uptake. Here, the maximal effect seen was at 10 µM resveratrol, with no further inhibition seen at 20 µM and 40 µM resveratrol. The time-course of the resveratrol effects on TSH induction of iodide uptake are shown in Figure 4B, where there was a transient resveratrol-induced increase in the iodide uptake after 6 h treatment. However, although this increased iodide uptake promoted by resveratrol was significant at the higher concentrations (20 µM and 40 µM resveratrol), it was lost by 24 h of resveratrol treatment, and furthermore, longer treatment with resveratrol resulted in a significant decrease in iodide uptake, after 48 h, regardless of the concentration used.

### Effects of resveratrol on thyroid radioiodine uptake in rats

The data presented above demonstrate that the treatment of the FRTL-5 thyroid cells with resveratrol induces a transient increase in NIS protein and function that is no longer evident after 24 h. Instead, treatments for longer times result in a significant decrease in NIS expression and function. Given these discrepancies, we investigated what effects the administration of resveratrol has *in vivo*.

**Figure 5:**
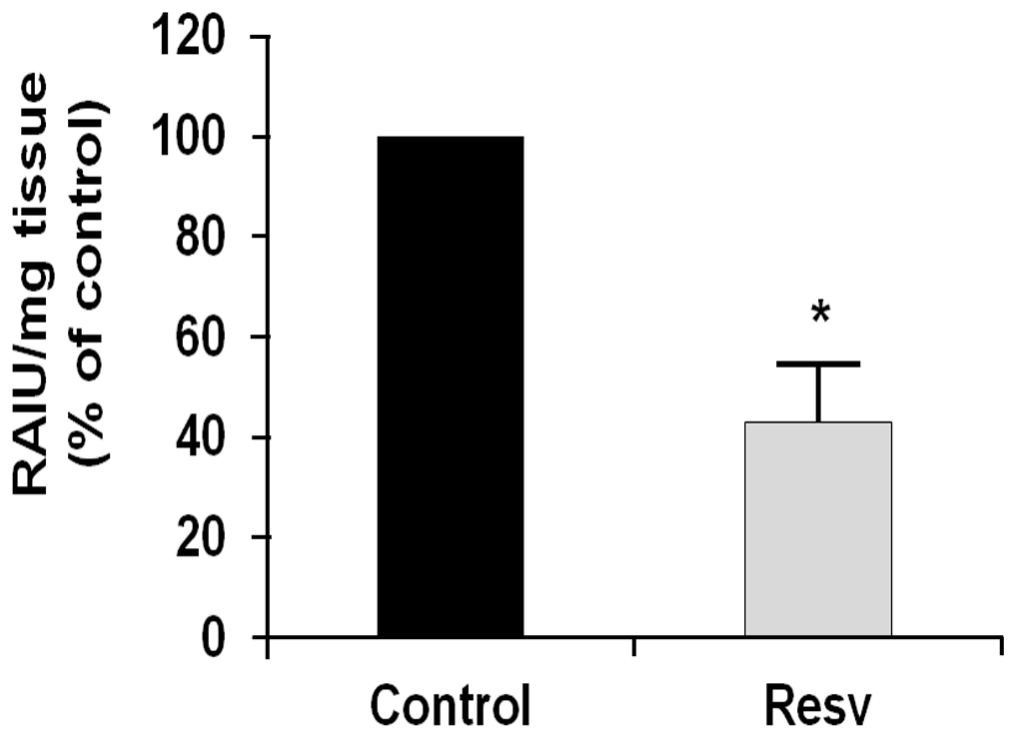
Effects of resveratrol on radioiodine uptake by the thyroid gland *in vivo*. Male Sprague-Dawley rats were treated with the control vehicle (Control, n = 6) or with 50 mg/kg/day resveratrol i.p. (Resv, n = 6), for 14 days. On the last day of treatment, the animals received [^125^I]-NaI (185 kBq i.p., each) 24 h prior to sacrifice. Their thyroids were removed and weighed, with their associated radioactivity (RAIU) determined in a gamma counter. Data (from iodide uptake per thyroid weight) are normalized means ±SD (control vehicle, 100%). *, p<0.05.

Here, 12 adult male Sprague-Dawley rats were divided in two groups (6/group). One group was treated with the control vehicle and the other with 50 mg/kg resveratrol i.p., for 14 days. These experimental conditions were based on the common use of the Sprague-Dawley breed to study resveratrol effects and metabolism [22]-[24]. On the last day of the resveratrol treatment, each rat received 185 kBq of Na[^125^I] i.p., and their thyroid radioiodide uptake was evaluated after 24 h. As shown in Figure 5, the treatment with resveratrol significantly decreased the thyroid radioiodide uptake, to 43%±7% of the control. These data provide evidence that resveratrol inhibits iodide uptake not only *in vitro*, but also *in vivo*.

**Figure 6:**
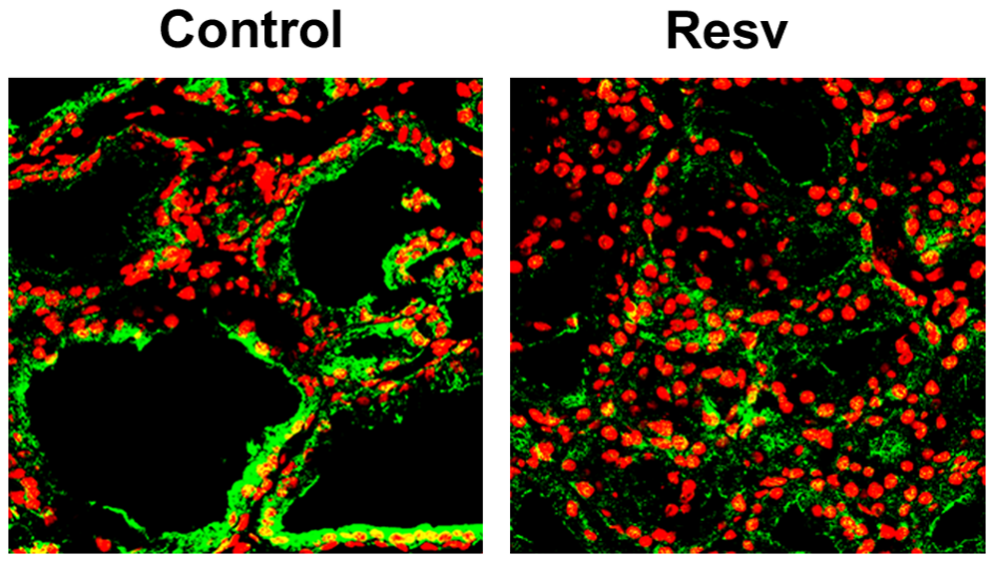
Effects of resveratrol on NIS protein expression in rat thyroid glands. Male Sprague-Dawley rats were treated with the control vehicle (Control, n = 4) or with 50 mg/kg/day resveratrol i.p. (Resv, n = 4), for 14 days. On day 15^th^, the animals were sacrificed and their thyroids were removed. Immunofluorescence analysis was performed using a mouse monoclonal anti-NIS antibody and an anti-mouse fluorescein-conjugated secondary antibody, Alexa Fluor 488, (green). Po-Pro-3 iodide was used to stain the nuclei (red). The negative control was performed using a mouse IgG preparations instead of the primary antibody (data not shown). The slides were visualized under a Zeiss LSM S10 confocal microscope with a x40 immersion lens. Representative data from four experiments are showed.

In line with the *in-vitro* data, treatment of rats with resveratrol resulted in down-regulation of the NIS protein in thyroid tissue, as evaluated by immunofluorescence (Figure 6).

We did not observed any significant effects of resveratrol on TSH and thyroid hormone concentrations (data not shown). We hypothesize that a longer treatment is required to affect TSH and thyroid hormone concentrations, due to the storage of preformed thyroid hormones in the gland. Further longer-term time course experiments are scheduled to provide further evidence towards this hypothesis.

## Discussion

In recent years, interest has grown around the potential health benefits of resveratrol, which has been postulated to be responsible for the so-called “French paradox”, the low incidence of cardiovascular diseases in the French population despite a diet rich in saturated fat [7], [25]. Indeed, several studies have shown that resveratrol can provide patient benefits against cancers and against cardiovascular, metabolic and inflammatory diseases. This has been proposed to be due to its anti-inflammatory, antioxidants and antiproliferative properties [1]–[5], [25]. As a consequence, there has been an increase in the use of resveratrol supplements and in the commercialization of resveratrol-based drugs or nutraceuticals. Furthermore, several clinical trials have been performed and are ongoing, to evaluate the effects of resveratrol on diabetes, cardiovascular abnormalities, and cancer [6], [7], [26], [27].

Several polyphenols, and particularly the flavonoids, have anti-thyroid activities [28], [29], although there are very few data available about the effects of resveratrol on thyroid function. To our knowledge, all of the studies performed to date, bar one, have been conducted in thyroid cancer cell lines [9]–[11] and have shown antiproliferative effects of resveratrol. Of particular interest, on the basis that FRTL-5 cells are a nontransformed cell line in continuous culture that represents a well-defined and reproducible *in-vitro* model of the thyroid gland [13]–[20], a study performed using these cells showed a transient increase in iodide uptake induced by resveratrol in the first 6 h to 12 h of treatment [12].

In the present study, we used these FRTL-5 cells, and we observed an inhibitory effect of resveratrol on NIS RNA as a function of time, with a significant decrease after 12 h of resveratrol treatment. Furthermore, we show here that the increase in NIS protein levels and iodide uptake that has previously been described [12] is a transient effect that is limited to the first 12 h of resveratrol treatment, and is replaced by an inhibitory effect when the treatment is prolonged beyond 24 h. At present, we do not have an explanation for the transient increase in the NIS protein and its function that is induced by resveratrol. However, as this effect is observed for the NIS protein levels and not for NIS gene RNA expression, this suggests that the transient increase in the NIS protein levels is the result of a post-transcriptional action of resveratrol on NIS metabolism. Indeed, post-transcriptional regulation of the NIS protein in thyroid cells has been demonstrated [21]. However, our data demonstrate that the main effect of resveratrol in FRTL-5 cells is the down-regulation of NIS expression and function. Recent data have shown that resveratrol can induce the expression of NIS in some thyroid carcinoma cell lines, such as ATC and FTC 133 cells, but not in other cell lines [10], [11]. These studies have indicated that there can be opposite effects of resveratrol on NIS expression in normal and cancer cells, and although this remains without explanation at present, it is a very interesting effect. Indeed, retinoic acid also showed a similar effects in regulating, in an opposite way, the expression of NIS in thyroid cancer cell lines, as compared to FRTL-5 cells [30]. Further investigations into the molecular mechanisms involved in this NIS gene regulation by resveratrol will be important to understand the dedifferentiation processes in thyroid cancer.

The discrepancy and complexity of the effects of resveratrol on NIS expression and function observed *in vitro* led us to evaluate the pharmacological effects of resveratrol *in vivo*. Sprague-Dawley rats were treated with 50 mg/kg/day resveratrol i.p. for 14 days. In a previous study, it was shown that this treatment provides a plasma concentration of resveratrol of about 10 µM [31], which is the same dose that used here in the *in-vitro* experiments, and that was effective in reducing NIS expression and function. Thus, an important point that needs to be emphasized here is that this dose of resveratrol that we used *in vivo* with these Sprague-Dawley rats is equivalent to a dose of about 9 mg/kg/day in human, according to dose translation from animal to human [32]. Indeed, in the use of resveratrol as a dietary supplement or nutraceutical, this dose is reached and even exceeded. Tablets that contain 300 mg to 500 mg resveratrol are available over the counter and through the internet. In human clinical trials, daily doses that range from 500 to 5000 mg/day resveratrol have been used, which have resulted in mean plasma resveratrol concentrations of 8.36 µg/L and 51.9 µg/L, respectively [26], [33].

Given these data, the results of the present study are particularly relevant, as we show here a significant 43% reduction in thyroid radioiodine uptake in the Sprague-Dawley rats, as compared to the control. Thus, the potential for untoward effects on iodide uptake should be considered in people who take high doses of resveratrol, and this would be particularly relevant in thyroid patients who are candidates for radioiodine treatment or thyroid scintigraphy. If the present data are confirmed in humans, resveratrol should be included among the substances that are banned prior to radioiodine administration or thyroid scintigraphy. Furthermore, the present data suggest the need to evaluate thyroid function in subjects who ingest large amounts of resveratrol. To the best of our knowledge, very few clinical trials have evaluated thyroid function in patients treated with resveratrol. In all of these studies, no adverse effects were observed regarding thyroid function, but the daily dose of resveratrol was relatively low, from 8 mg/day to 16 mg/day [7].

In conclusion, our data show that resveratrol is an inhibitor of NIS gene expression and function. This effect is here demonstrated *in vitro* and is confirmed by the inhibition of thyroid radioiodine uptake and NIS protein expression in normal rats. These data are relevant given the worldwide diffusion of dietary supplements that contain high amounts of resveratrol, and the great interest in both the general and scientific communities for the use of resveratrol as a therapeutic agent [6], [34].

Thus, human studies are needed to confirm the effects of resveratrol on iodide uptake and to evaluate the potential therapeutic use of resveratrol in hyperthyroidism. Meanwhile, we suggest that the use of resveratrol is avoided in pregnant and lactating women, and in thyroid patients with hypothyroidism, or who are candidates for radioiodine administration.
